# Physiologic Decompression of Lumbar Spinal Stenosis Through Anatomic Restoration Using Trans-Kambin Oblique Lateral Posterior Lumbar Interbody Fusion (OLLIF): A Retrospective Analysis

**DOI:** 10.7759/cureus.11716

**Published:** 2020-11-26

**Authors:** Hamid Abbasi

**Affiliations:** 1 Ambulatory Surgical Clinic, Tristate Brain and Spine Institute, Alexandria, USA; 2 Neurosurgery, Inspired Spine Health, Minneapolis, USA

**Keywords:** lumbar fusion, spine, new technology in spine surgery, minimally invasive and robotic spine surgery, lumbar spinal stenosis (lss), spinal fusion

## Abstract

Introduction

Lumbar spinal stenosis (LSS) is one of the most common indications for spinal surgery. Traditionally, decompression is achieved by removing bony and ligamentous structures through open surgery. However, recent studies have shown that symptomatic relief can be accomplished in many patients by increasing intervertebral and interpedicular height using fusion alone. In this study, we evaluate whether trans-Kambin oblique lateral lumbar interbody fusion (OLLIF) can effectively and safely relieve symptoms of LSS when an indication for fusion is present.

Methods

This is a retrospective single surgeon cohort study of 187 patients with LSS who underwent 189 OLLIF procedures between 2012 and August 2, 2019. Inclusion criteria for this study were age >18 years with symptoms of LSS, including pain, sensory, and motor deficits, and an additional indication for fusion, which included spondylolisthesis, degenerative disk disease, disk herniation, or scoliosis. Exclusion criteria were the bony obstruction of the approach, osteogenic spinal canal stenosis, large facet hypertrophy, and listhesis grade II or greater. The primary outcome was a change in the Oswestry Disability Index (ODI) one year after surgery. Secondary outcomes were the resolution of radiculopathy at the first follow-up visit and one year after surgery, complication rates, surgery time, blood loss, and hospital stay.

Results

ODI improved from 52% pre-op to 37% at the one-year follow-up. At the first follow-up, radiculopathy had resolved in 39% of patients, and 72% of patients experienced improvement of 50% or greater. One year after surgery, radiculopathy had resolved in 52% of patients and 74% experienced improvement of 50% or greater. Single-level surgeries required 56.4±21.5 minutes, with a mean hospital stay of 1.6^‑^±2.4 days. Nerve irritation occurred in 12% of patients at the first postoperative follow-up and persisted in 6.8% of patients one year after surgery. There was one case each of persistent weakness at one year, infection, and cage subsidence.

Conclusion

Trans-Kambin OLLIF delivers anatomic restoration of intradiscal and interpedicular distance, which results in physiologic decompression of lumbar spinal stenosis in patients undergoing lumbar fusion for degenerative or herniated disk disease, spondylolisthesis, or scoliosis. Amongst patients with LSS, OLLIF results in significant improvement of radiculopathy and patient-reported disability in the majority of patients with low rates of long-term complications. Unlike other minimally invasive surgery (MIS) fusions, OLLIF can be safely used from T12-S1.

## Introduction

Low back pain is one of the most prevalent and expensive health conditions in the Western world, with up to 80% of all people suffering from it at some point during their life [1-2]. One of the most common indications for surgery among patients with low back pain is lumbar spinal stenosis (LSS) [3]. LSS most commonly involves the compression of the spinal nerve or nerve roots arising from degenerative changes in the lumbar spine. The traditional surgical approach for LSS is the decompression of the neural structures using foraminotomy, laminotomy, or laminectomy. Decompression is sometimes complemented with posterior or interbody fusion, in which the vertebrae above and below the stenotic spinal segment are fused together. Fusion is typically indicated when other spinal conditions, such as spondylolisthesis or scoliosis, are present and causing disabling symptoms but can also be indicated to treat iatrogenic instability [4]. The exact choice of the procedure remains controversial, as traditional fusion has not been shown to consistently improve outcomes, although studies comparing decompression alone to fusion with decompression have traditionally been conducted using open, instead of minimally invasive, surgery [5-6].

The most common surgical approach is transforaminal lumbar interbody fusion (TLIF), which involves cutting through the paraspinal muscles, the ligaments that stabilize the spine, and resection of the facet joint capsule [7]. Alternative minimally invasive surgery (MIS) approaches to lumbar fusion have recently become popular, including lateral lumbar interbody fusion (LLIF) and oblique lateral lumbar interbody fusion (OLLIF) [8-9]. However, these approaches do not involve bone removal or direct decompression of neural elements because they do not involve direct visualization of these structures. Instead, these procedures improve function by restoring the height of the disk space and neural foramen [10]. This effect has been called “indirect” decompression, but we prefer to refer to it as anatomical restoration resulting in physiologic decompression. We believe that “indirect” vs. “direct” decompression creates a false dichotomy between different types of procedures based on the surgical approach. Realistically, the outcome that matters to patients is physiologic decompression resulting in symptom relief. This relief can be achieved by removing bone using laminectomy and foraminotomy; however, it can also be achieved by the anatomical restoration of intradiscal and interpedicular distance without the added trauma involved in resecting bones and ligaments. Accessing these structures, in turn, requires passage through muscle tissue, resulting in devascularization of the bone and denervation of the muscle, which may transform these functional structures into functionally inert scar tissue.

Physiological decompression through anatomical restoration has been previously shown to be an effective treatment for lumbar spinal stenosis in several trials evaluating LLIF for this purpose [11-12]. However, LLIF is a difficult procedure for L4-5 and L5-S1, the levels that account for the majority of lumbar pathology. In this study, we evaluate the performance of oblique lateral lumbar interbody fusion (OLLIF) in patients with symptomatic LSS. OLLIF has previously been shown to be a safe and effective fusion of the lumbar spine. In OLLIF, the disk is approached from approximately 45° posteriorly and accessed through Kambin’s triangle [13-14]. Here, we report our experience of 189 patients with LSS who underwent OLLIF.

## Materials and methods

Study design

This study is a retrospective cohort study of 187 patients with LSS who underwent 189 OLLIF procedures between 2012 and August 2, 2019. Procedures were performed by a single surgeon in seven hospitals in Minnesota. Institutional review board (IRB) exemption was granted by Pearl Pathways IRB 7/29/2020. Inclusion criteria for this study were patients >18 years who had symptomatic degenerative LSS with another indication for fusion. Indications for fusion included spondylolisthesis, degenerative disk disease, disk herniation, and scoliosis. All patients underwent preoperative imaging, including a combination of magnetic resonance imaging, X-ray of the lumbar spine with flexion and extension, discogram, and computed tomography (CT) scan. Stenosis was diagnosed based on the presence of sensory deficit, weakness, claudication, radiculopathy, and imaging findings at the discretion of the principal investigator. Exclusion criteria were the bony obstruction of the approach, osteogenic spinal canal stenosis, large facet hypertrophy, and grade II or greater listhesis. All patients underwent and failed at least six months of conservative therapy, including physical therapy, therapeutic injections, bracing, and behavioral modifications prior to surgery.

The OLLIF procedure

**Figure 1:**
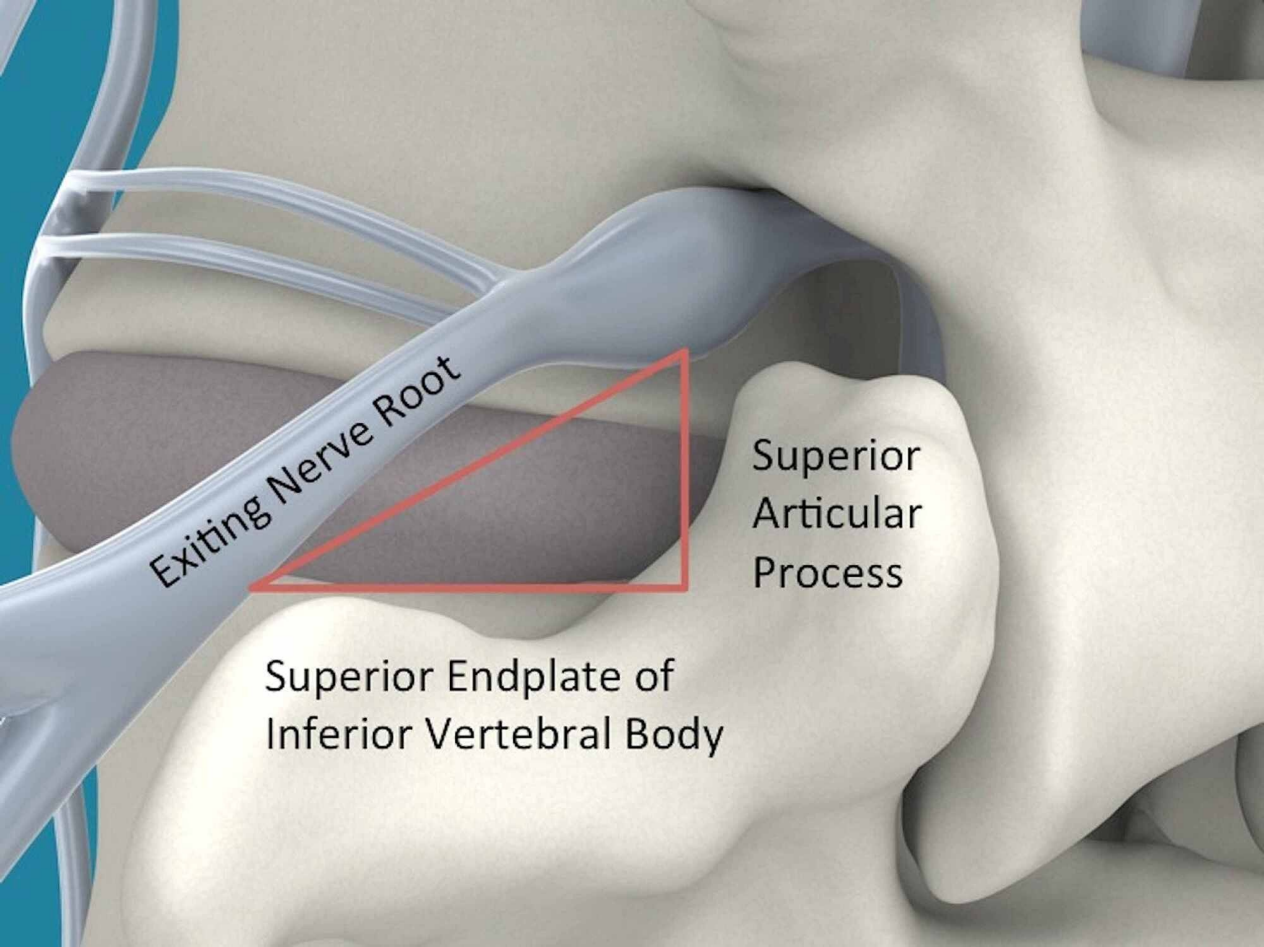
Anatomy of Kambin's triangle Kambin's triangle consists of the superior endplate of the caudal vertebral body, the exiting nerve root, and the superior articular process

**Figure 2:**
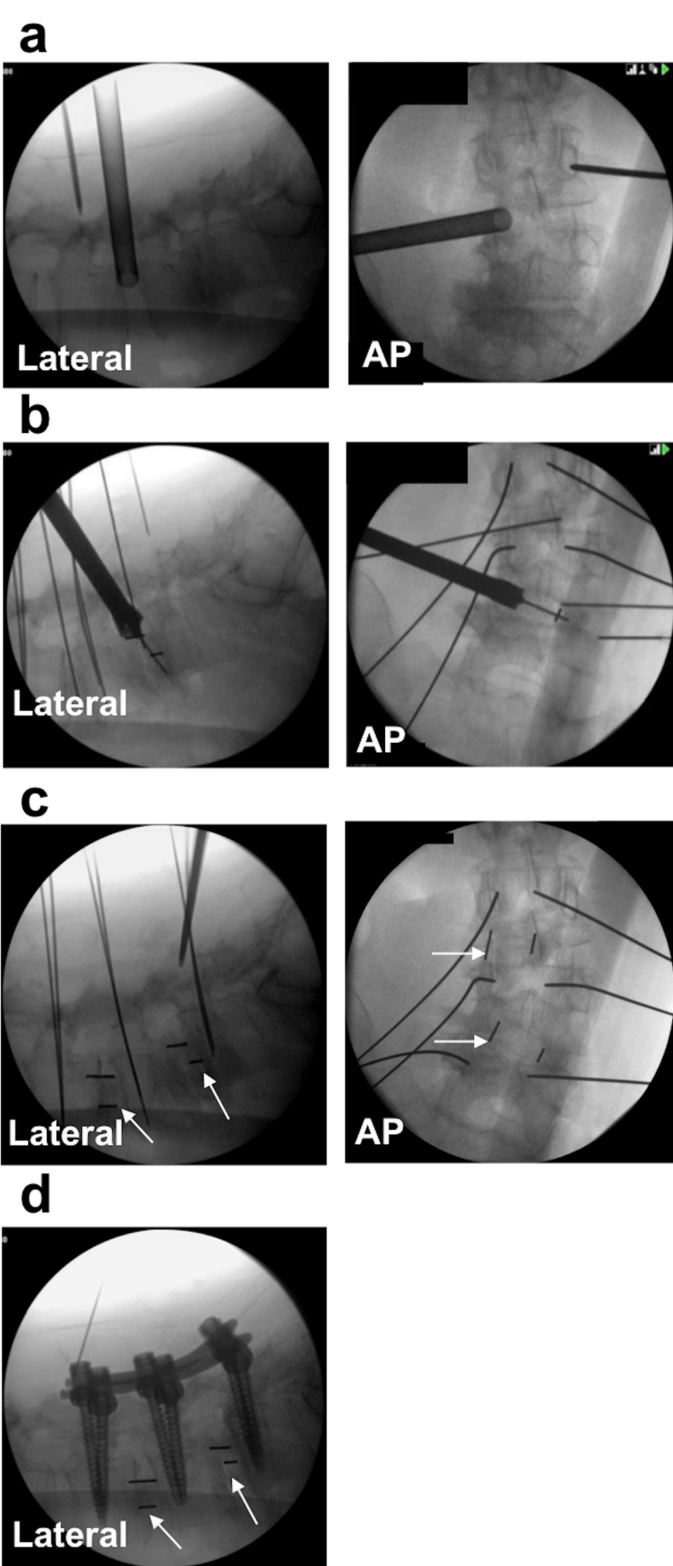
The steps of the OLLIF procedure under bilateral fluoroscopy a. The disk is approached guided by fluoroscopy and electrophysiological monitoring. b. The cage is inserted. c. The interbody fusion is complete, arrows indicate the locations of cages. d. OLLIF is complemented with percutaneous posterior pedicle screw fixation. Figure reproduced with permission from reference [8]. OLLIF: oblique lateral lumbar interbody fusion

The OLLIF procedure is based on a trans-Kambin approach to the disk space, and we have previously described the steps of the operation in detail [8,13]. Kambin's triangle consists of the superior endplate of the caudal vertebral body, the exiting nerve root, and the superior articular process (Figure 1). The operation is performed in the prone position, assisted by anterior-posterior (AP) fluoroscopy, lateral fluoroscopy, and electrophysiological monitoring. The disk space is accessed through Kambin's triangle using a blunt probe. By stimulating the probe up to 4 mA, we verify that a safe corridor has been established, which is subsequently dilated until a 10 mm access portal can be placed. Discectomy is performed through the access portal and tricalcium phosphate soaked in autologous bone marrow aspirate is packed into the disk space. Subsequently, the cage is placed under fluoroscopic guidance. The entire procedure is performed percutaneously without direct visualization. OLLIF is complemented with posterior pedicle screw fixation to achieve 360° fusion [15]. The steps of OLLIF under fluoroscopy are shown in Figure 2, Video 1, and Video 2.

**Video 1:** Lateral fluoroscopic view of physiologic decompression through anatomic restoration of the intradiscal and interpedicular distance using trans-Kambin OLLIF OLLIF: oblique lateral lumbar interbody fusion

**Video 2:** AP fluoroscopic view of physiologic decompression through the anatomic restoration of the intradiscal and interpedicular distance using trans-Kambin OLLIF AP: anterior-posterior; OLLIF: oblique lateral lumbar interbody fusion

Outcome measures and analysis

Patient age, gender, body mass index (BMI), and indications were recorded at the preoperative clinic visit and entered into a custom database. Operating room staff recorded skin to skin surgery time, fluoroscopy time, and blood loss, which was calculated by weighing sponges because no suction is used. The hospital stay was recorded upon discharge, and all perioperative data were transferred to a custom database. Wound infections, cage subsidence, and neurologic complications were recorded at follow-up visits. Neurologic complications were classified as nerve irritation if paresthesia, dysesthesia, or mild weakness down to 4+/5 were noted on physical exam. Neuropraxia was defined as a weakness of 4/5 or worse. Neurologic complications were only counted if they corresponded to a level of surgery, were not present prior to surgery, and appeared within the immediate postoperative period. Early complications occurred within 90 days of surgery; late complications were those still present 300 days after surgery. Patient-reported outcomes were measured using the modified Oswestry Disability Index (ODI) [16]. We included the most recent ODI prior to the operation, provided it was completed within one year of the operation. Postoperatively, we included the first ODIs completed more than 300 days after the operation, which was typically at the one-year follow-up appointment. The subjective change in radiculopathy corresponding to the level of surgery was assessed by clinical exam and classified into the following categories: radiculopathy gone completely, radiculopathy gone at rest but provoked by activity, significant improvement in radiculopathy (decrease ≥50%), some decrease in radiculopathy (decrease <50%), no change or worsening radiculopathy, or unable to assess from visit documentation. Data were collected in real-time, placed in a custom database, and exported for analysis and visualization in R3.4.

## Results

Demographics of the study population along with indications for surgery are displayed in Table 1. There were 189 patients in our study with a mean age of 63±14 and a mean BMI of 31.9±6.9. Perioperative outcomes are listed in Table 2. Blood loss averaged 84.8±81 ml. Mean hospital stay was 1.8±1.9 days. Skin to skin surgery time ranged from 56.4±21.5 minutes for one-level procedures to 159.5±62.7 min for operations involving four or more levels.

**Table 1:** Study group characteristics and indications BMI: body mass index; SD: standard deviation

N	189
BMI (mean (SD))	31.9 (6.9)
Age (mean (SD))	63.4 (13.7)
Degenerative or herniated disk = 1 (%)	158 (83.6)
Scoliosis = 1 (%)	32 (16.9)
Spondylolisthesis = 1 (%)	83 (43.9)
Foraminal Stenosis = 1 (%)	55 (29.1)

**Table 2:** Perioperative outcomes Surgery time was measured skin to skin; SD: standard deviation

Levels	1	2	3	4+	Overall
N	53	76	50	10	189
Blood loss (ml) (mean (SD))	49.7 (61.1)	73.7 (64.3)	120.6 (82.4)	176.2 (140.9)	84.8 (81.1)
Surgery time (min) (mean (SD))	56.4 (21.5)	68.1 (15.5)	99.5 (36.1)	159.5 (62.7)	78.1 (37.6)
Fluoroscopy time (s) (mean (SD))	182.2 (75.7)	307.0 (126.9)	431.5 (177.8)	660.4 (319.5)	323.7 (190.0)
Hospital stay (days) (mean (SD))	1.6 (2.4)	1.9 (1.7)	1.5 (1.5)	4.0 (1.6)	1.8 (1.9)

Patient-reported disability on the ODI improved from 52% pre-op to 37% at the one-year follow-up (Table 3), with significant improvements in all categories of the ODI. Radiculopathy, as assessed by physical exam, improved in the majority of patients (Table 4). At the first postoperative visit, radiculopathy was completely gone in 39% of patients and 72% of patients experienced at least significant improvement in radiculopathy, defined as an improvement of 50% or greater. This improvement in radiculopathy was durable at one year when 52% of patients experienced complete resolution of radiculopathy and 74% experienced significant improvement.

**Table 3:** Pre- and postoperative Oswestry Disability Index scores Preop was defined as within one year prior to surgery; postop was at least 300 days after surgery SD: standard deviation

	Pre-op	Post-op	p
Number of patients	103	105	
Pain (mean (SD))	3.52 (1.35)	2.16 (1.72)	<0.001
Care (mean (SD))	2.12 (1.30)	1.36 (1.34)	<0.001
Lifting (mean (SD))	3.19 (1.15)	2.82 (1.43)	0.038
Walking (mean (SD))	2.86 (1.06)	2.12 (1.45)	<0.001
Sitting (mean (SD))	1.97 (1.32)	1.20 (1.01)	<0.001
Standing (mean (SD))	3.08 (1.31)	2.39 (1.56)	0.001
Sleeping (mean (SD))	2.11 (1.37)	1.58 (1.34)	0.006
Social (mean (SD))	2.68 (1.53)	1.66 (1.56)	<0.001
Traveling (mean (SD))	2.07 (1.21)	1.52 (1.19)	0.001
Housework (mean (SD))	2.44 (1.40)	1.87 (1.30)	0.003
Score (mean (SD))	52.08 (17.18)	37.37 (21.35)	<0.001

**Table 4:** Change of radiculopathy from preoperative baseline in the early and late postop period Early was defined as ≤90 days after surgery and late follow-up was defined as ≥300 days after surgery. Percentages were calculated as a percentage of those who were seen at follow-up.

	Early	Late
Number of patients evaluated	147	123
Complete resolution (N (%))	57 (38.8)	64 (52)
Resolved at rest, provoked by activity (N (%))	9 (6.1)	8 (6.5)
≥50% improvement (N (%))	40 (27.2)	19 (15.4)
<50% improvement (N (%))	6 (4.1)	9 (7.3)
No improvement (N (%))	13 (8.8)	13 (10.6)
Unable to assess (N (%))	22 (15)	10 (8.1)

Complications are listed in Table 5. The most common complication was nerve irritation, which was present in 12% of patients at the first postoperative follow-up and persisted in 6.8% of patients one year after surgery. Neuropraxia was present in 2.5% of patients immediately postoperatively but only persisted at one year in a single patient (0.8%). The only other complications were one case of superficial wound infection not requiring reoperation and one case of cage subsidence.

**Table 5:** Early and late complications Early is defined as ≤90 days after surgery; late is defined as ≥300 days after surgery. Percentages are calculated as a percentage of patients who completed follow-up at each timepoint.

Complication	Early	Late
Number of patients evaluated	158	132
Nerve irritation (N (%))	19 (12)	9 (6.8)
Neuropraxia (N (%))	4 (2.5)	1 (0.8)
Superficial infection (N (%))	1 (0.6)	0 (0)
Cage subsidence (N (%))	0 (0)	1 (0.8)

## Discussion

In this study, we demonstrate that OLLIF can effectively relieve symptoms of LSS when another indication for surgery is present. Our study adds to a series of recent trials that show that the anatomical restoration of disc height and interpedicular distance can result in physiologic decompression, as proven by the improvement of symptoms of LSS [11,17]. Physiologic decompression has several advantages over direct decompression because it can be performed in an MIS manner. Although several MIS options exist, OLLIF has several advantages over other procedures because it can be performed from T12-S1, and, with some modification, can be performed in the thoracic spine below T6 and requires less surgery time than other MIS fusions [18].

**Figure 3:**
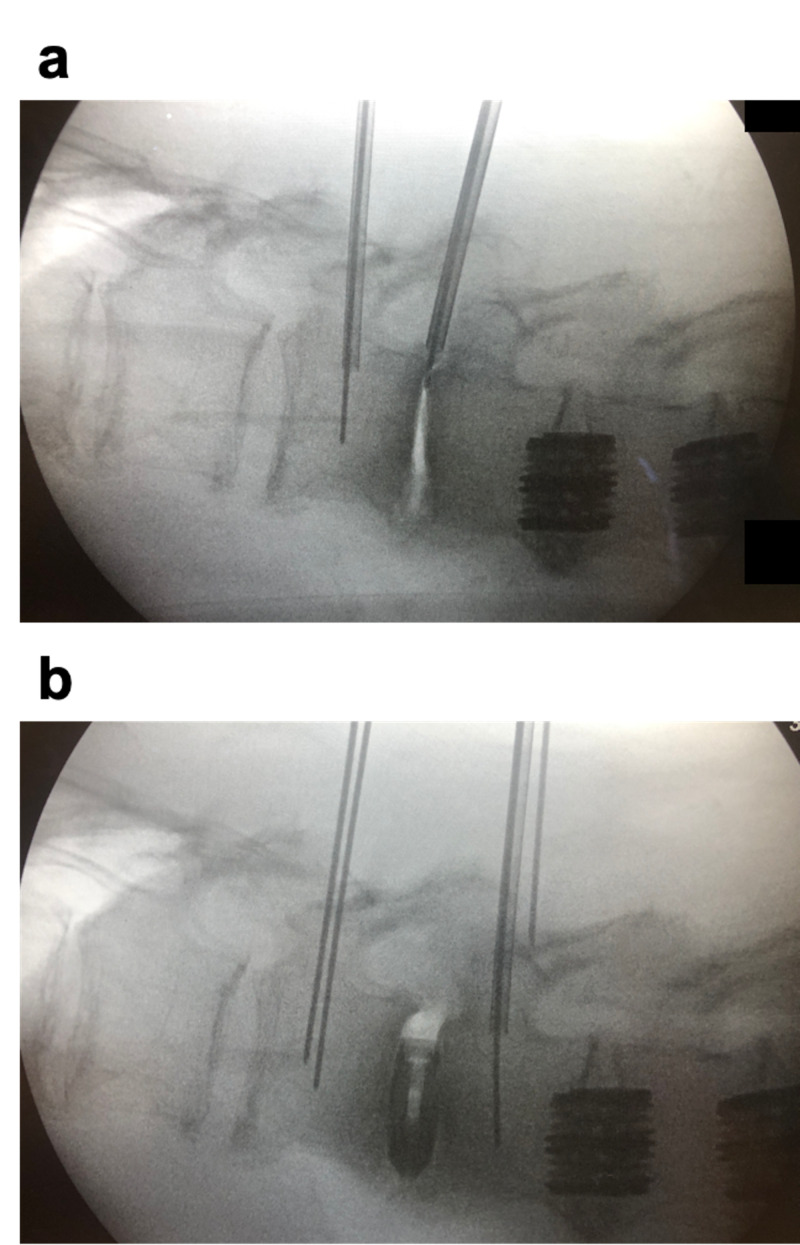
Lateral fluoroscopy demonstrating the restoration of the disk and foraminal height by a large interbody cage a) pre-cage placement; b) post-cage placement

We demonstrated that the majority of patients with LSS who underwent OLLIF had significant improvement in their radiculopathy. Amongst patients who were evaluated at the first follow-up visit, 72% reported at least significant improvement in their radiculopathy. This improvement was sustained, with 74% of patients reporting at least significant improvement one year after surgery. These results indicate that disk height restoration results in physiologic decompression (also known as “indirect” decompression). This finding is consistent with previous studies that indicate indirect decompression is effective at relieving symptoms of neurogenic claudication. The mechanism of physiologic decompression is thought to relate to increased foraminal and intervertebral disk heights. As shown in Figure 3, Video 1, and Video 2, we can achieve significant increases in foraminal size because OLLIF allows for the placement of large cages. A recent study on patients with spondylolisthesis found that as compared to direct decompression using MIS TLIF, LLIF resulted in greater increases in foraminal height but smaller increases in canal dimensions [19]. However, one disadvantage of physiologic decompression is that it is unlikely to benefit patients with osteogenic canal stenosis or thickened ligamentum flavum. We, therefore, hypothesize that OLLIF is more likely to improve symptoms of radiculopathy than neurogenic claudication. In these patients, when fusion is indicated, we prefer a staged approach with OLLIF followed by direct decompression using MIS hemilaminectomy, a procedure in which we achieve bilateral decompression through a unilateral approach without the removal of the whole lamina or facets.

**Figure 4:**
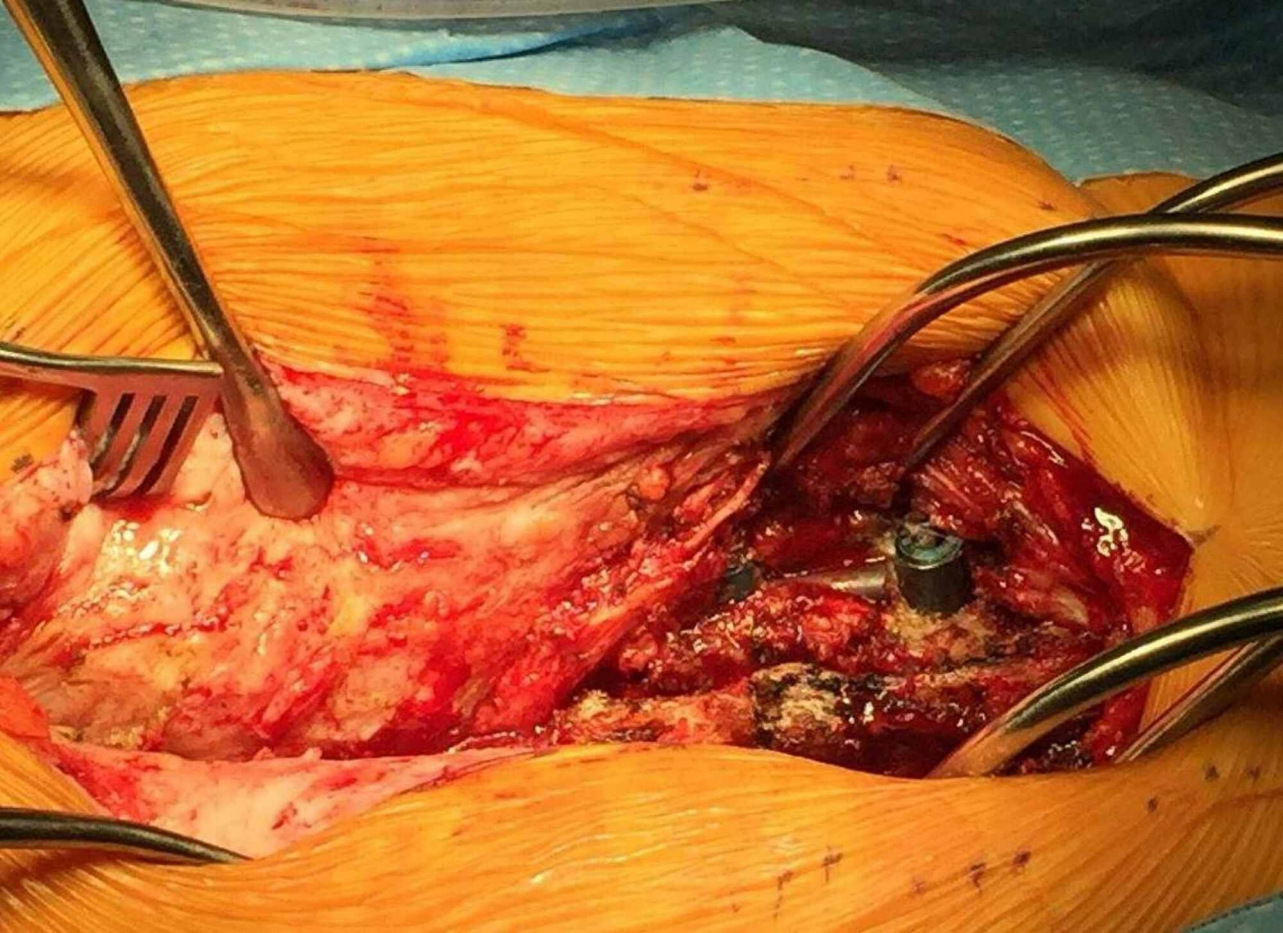
Follow-up surgery on a patient who had previously undergone L3-L5 TLIF (right) followed by L1-2 OLLIF (left) This patient had L3-5 TLIF (right side of image), followed by L1-2 OLLIF (left side of image). The patient subsequently suffered trauma requiring open surgery. TLIF results in significant scar tissue formation of the paraspinal soft tissue, whereas OLLIF leaves these tissues well vascularized. TLIF: transforaminal lumbar interbody fusion; OLLIF: oblique lateral lumbar interbody fusion

Physiologic decompression using OLLIF offers several advantages over direct decompression. We have previously shown that OLLIF has a much shorter surgery time and hospital stay as compared to TLIF, which reduces the cost of surgery [8,13,20]. With direct decompression, the direct visualization of neural structures is essential to prevent nerve injury and achieve decompression of the target anatomy. This approach requires larger incisions and more dissection of the paraspinal soft tissue than in OLLIF. This may increase postoperative pain, infection rates, and the incidence of adjacent segment disease [21]. In this study of 189 operations, we had only a single case of superficial wound infection effectively managed with oral antibiotics. In a recent procedure involving a patient who had undergone both TLIF and OLLIF, we found significantly improved vascularization of the paraspinal muscle after OLLIF as compared to TLIF (Figure 4), which may result in a decreased rate of wound infection and increased rates of fusion because the blood supply to the posterior spine comes from perforators through the muscle. Despite the fact that neural structures are not directly visualized in OLLIF, we report complication rates that were similar to those reported for open procedures, although it is difficult to compare these rates because neurologic complications are not coded in a uniform fashion. At the one-year follow-up, we report a 7% rate of persistent nerve root irritation and only one case (0.8%) of persistent neuropraxia. A recent review of TLIF procedures found neurologic complication rates of 5% with MIS-TLIF and 3% with open TLIF [22]. Patient-reported outcomes are similar for OLLIF as compared to procedures providing direct decompression. In this study group, ODI improved by 15 points at the one-year follow up; this compares to a mean improvement of 16 points after direct decompression in a recent large registry study [23].

OLLIF has several advantages over other MIS approaches to the lumbar spine such as LLIF and OLIF-ATP. In this study, single-level fusions with posterior instrumentation required, on average, 56 minutes skin to skin, which is much faster than any other fusion surgery reported in the literature today [24-25]. We have also demonstrated that unlike MIS-TLIF, obesity does not increase the time or difficulty of OLLIF, allowing patients who would otherwise be poor surgical candidates to undergo fusion [26]. Finally, OLLIF can be safely performed from T12-S1. Other approaches, such as LLIF and OLIF-ATP, are very difficult or impossible at L5-S1 due to the position of the iliac crest [27-28].

The main weakness of this study is the retrospective nature of the analysis and the lack of a control group. This is a single-surgeon study with a community practice in the Midwest, and the results may not generalize to other surgeons and practice settings. The diagnosis of LSS was based on the judgment of the surgeon because LSS is a clinical diagnosis. Additionally, the practice in this study is a combination of urban and rural sites, which often requires patients to travel long distances. Therefore, follow-up rates are relatively low in this study and true rates of complications may be higher than reported. In our subjective experience, patients who are doing well are less likely to present for follow-up appointments because they do not think it is necessary to travel a long distance for a clinic visit. We do not present any radiologic measurements, such as foraminal height, because these were not recorded in a uniform fashion. Therefore, we cannot draw any conclusions about the association of any particular anatomic change with symptomatic improvement.

## Conclusions

Trans-Kambin OLLIF can result in anatomic restoration of intradiscal and interpedicular distance, which results in the physiologic decompression of lumbar spinal stenosis in patients undergoing lumbar fusion for degenerative or herniated disk disease, spondylolisthesis, or scoliosis. Amongst patients with LSS, OLLIF results in significant improvement of radiculopathy and patient-reported disability in the majority of patients with low rates of long-term complications.
